# Tips and tricks for the assembly of a *Corynebacterium pseudotuberculosis* genome using a semiconductor sequencer

**DOI:** 10.1111/1751-7915.12006

**Published:** 2012-12-02

**Authors:** Rommel Thiago Jucá Ramos, Adriana Ribeiro Carneiro, Siomar de Castro Soares, Anderson Rodrigues dos Santos, Sintia Almeida, Luis Guimarães, Flávia Figueira, Eudes Barbosa, Andreas Tauch, Vasco Azevedo, Artur Silva

**Affiliations:** 1 Institute of Biological Sciences, Federal University ParáBelém, Pará, Brazil; 2 Institute of Biological Sciences, Federal University Minas GeraisBelo Horizonte, Minas Gerais, Brazil; 3 Center for Biotechnology, Institute for Genome Research and Systems BiologyBielefeld, Germany

## Abstract

New sequencing platforms have enabled rapid decoding of complete prokaryotic genomes at relatively low cost. The Ion Torrent platform is an example of these technologies, characterized by lower coverage, generating challenges for the genome assembly. One particular problem is the lack of genomes that enable reference-based assembly, such as the one used in the present study, *Corynebacterium pseudotuberculosis* biovar equi, which causes high economic losses in the US equine industry. The quality treatment strategy incorporated into the assembly pipeline enabled a 16-fold greater use of the sequencing data obtained compared with traditional quality filter approaches. Data preprocessing prior to the *de novo* assembly enabled the use of known methodologies in the next-generation sequencing data assembly. Moreover, manual curation was proved to be essential for ensuring a quality assembly, which was validated by comparative genomics with other species of the genus *Corynebacterium*. The present study presents a *modus operandi* that enables a greater and better use of data obtained from semiconductor sequencing for obtaining the complete genome from a prokaryotic microorganism, *C. pseudotuberculosis*, which is not a traditional biological model such as *Escherichia coli*.

## Introduction

With the advent of next-generation sequencer (NGS) platforms, such as 454 Roche, SOLiD and Illumina, there has been an increase in the number of projects for whole-genome sequencing (WGS) mainly due to cost reduction and the increased speed of sequencing and data generation. Nonetheless, problems related to the assembly of sequences arising from these platforms, such as the resolution of repetitive regions of the genome and representation of low-coverage regions with short reads, have made the development of hybrid strategies with different platforms and assemblers necessary to achieve successful assemblies (Kircher and Kelso, 2010; Cerdeira *et al*., 2011).

Currently, these technologies are being developed and others are emerging, such as the Ion Torrent sequencer. The Ion Torrent identifies nucleotides by using a semiconductor to detect the pH change caused by the release of H^+^ protons after a nucleotide is incorporated into the sequence, with each nucleotide added in a different cycle (Rothberg *et al*., 2011). In this platform, sequencing coverage varies according to the chip used: 314, 316 and 318 chips are capable of producing 10 Mb, 100 Mb and 1 Gb, respectively, of sequences with an average read length of 200 bp (http://www.appliedbiosystems.com). Therefore, the Ion Torrent can be used to sequence the genomes of prokaryote organisms in a fast, low-cost manner.

*Corynebacterium pseudotuberculosis* is included in the CMNR group, which includes bacteria of the genera *Corynebacterium*, *Mycobacterium*, *Nocardia* and *Rhodococcus*. These bacteria are of interest in veterinary science, and their high lipid content, including mycolic acid, the most prevalent, from the cell wall and meso-diaminopimelic acid distinguishes them from other genera. Polysaccharides, such as arabinose, galactose and some types of mannose, can also be found. As demonstrated in other studies, mycolic acid is the best characterized component and plays an important role in the virulence of *Mycobacterium tuberculosis* (Dorella *et al*., 2006).

*Corynebacterium pseudotuberculosis* infection leads to significant economic losses related to the decrease in productivity of infected animals. Various strains of *C. pseudotuberculosis* from goats and sheep (biovar ovis) have already been isolated, sequenced and studied, including strains 1002 and C231 (Ruiz *et al*., 2011). However, there is still a scarcity of genomic information on strains isolated from horse (biovar equi), which causes significant problems for horse breeders in California (USA) (Doherr *et al*., 1998).

The present study reports the assembly of the complete genome sequence of *C. pseudotuberculosis* 316, isolated from a horse and sequenced using the Ion Torrent platform. A pipeline was created for genome assembly that consisted of a new tool with a quality filter and in-house scripts for data preprocessing and assembly software used for short reads without the expected requirement of algorithm optimization (Earl *et al*., 2011). The functional annotation of the genome was subsequently performed, followed by a comparative analysis between the pathogenicity islands (PAIs) identified in strain 316 and other *C. pseudotuberculosis* strains already deposited in the biological databases.

## Results and discussion

### Contigs generated by the *de novo* assembly

**Figure 1:**
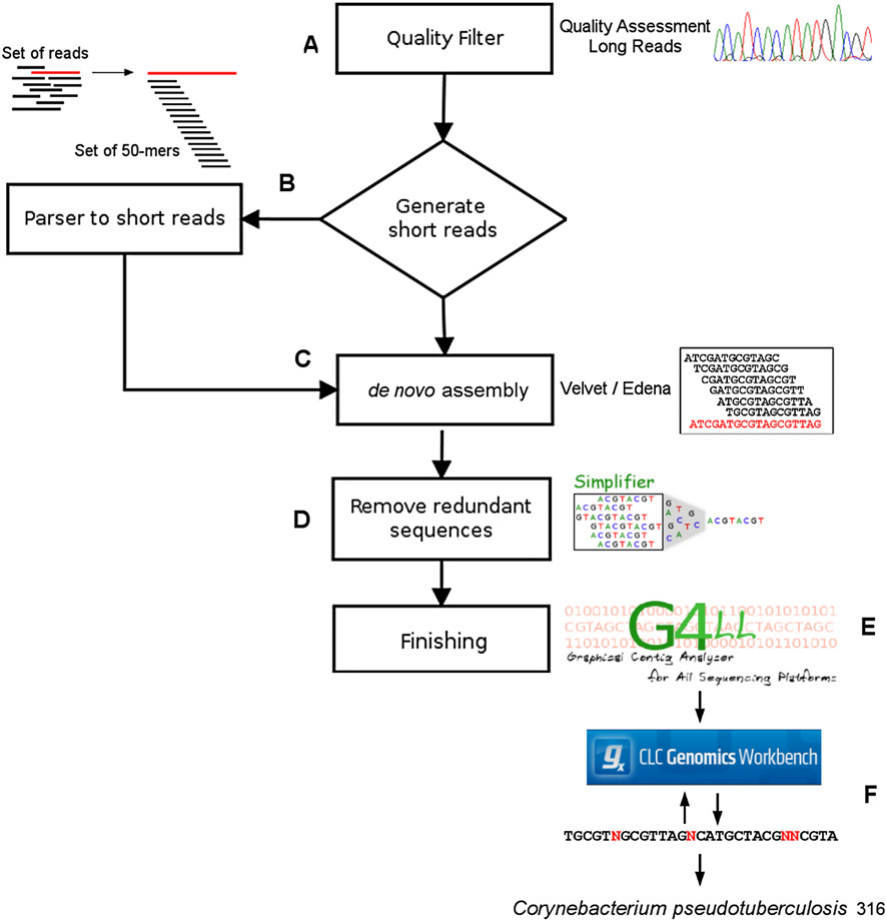
Workflow representing the assembling process and each step for the generation of a consensus sequence along with their receptive software/methods. The assembling process consists of: (A) data treatment, where the reads are trimmed and removed when the mean quality value on the region does not reach the cut-off value; (B) the filtered reads are then fragmented into short reads with the same size to be submitted to *de novo* assembly; (C) *de novo* assembly using diverse parameters and softwares; (D) removal of redundant sequences by the Simplifier software; (E) extending of sequences with similar extremities using the software G4ALL and the genome of a related species as reference; and (F) recursive analyses based on short-read alignments, against a preliminary scaffold, using CLC, gap identification using an *in-house* script and manual curation of gaps/frameshifts.

The three chips used in sequencing yielded a total of 898 389 reads (160 607 819 bp). These data were submitted to the first stage of the pipeline (Fig. 1): quality filter with Quality Assessment software, long-read version, to achieve the maximum quality of sequences possible. After this step, 443 632 reads (37 247 006 bp) remained, which represented 16 × sequence coverage. If the quality filter had been based solely on the average quality of the reads, there would have been only 16 467 reads (1 918 221 bp) remaining, which would represent less than 1 × coverage of the genome.

In the second step, the filtered reads were subjected to assembly using the Velvet and Edena3 software programs. The best assemblies with Velvet and Edena3 were obtained using a k-mer of 31 and coverage cut-off of 5 and k-mer of 45 and coverage cut-off of 6, respectively, and the largest N50 was observed with the Velvet assembly (Table 1).

**Table 1:** Quality evaluation of Velvet-and Edena-assembled genomes

	N50	Largest contig	Smaller contig	Number of contigs	Bases
Velvet	423	2476	100	7 260	2 482 519
Edena3	210	1109	100	12 471	2 370 369

Once the best results from Velvet and Edena were combined, 19 731 contigs were obtained (N50 of 295). However, after the removal of redundant contigs by the Simplifier software (http://sourceforge.net/projects/simplifier), only 16 287 contigs remained (N50 of 323), resulting in a 17.45% reduction in the number of contigs.

### Assembly completion

When processing the 16 287 contigs in G4ALL (http://g4all.sourceforge.net/), using the genome of *C. pseudotuberculosis* FRC41 as a reference (Trost *et al*., 2010), 160 contigs that aligned in more than one region of the genome, 20 that had non-specific alignments (length < 40 bp and *E*-value > 1 × 10^−5^), 629 that did not align and 15 478 that were mapped to unique regions were identified. Similarities were present between the extremities of 1222 of these contigs, which were therefore extended. Only 14 256 contigs that were solely mapped to the reference genome remained.

Contigs mapped against the reference genome using G4ALL (14 436 sequences), even when below the cut-off criteria, were used in the CLC Genomics Workbench 4.7.2 software for alignment against the FRC41 genome. After this alignment, only 312 sequences were not mapped (55 kb), and a primary scaffold was generated with 5758 gaps (3687 of 1 bp, 1003 of 2 bp, 1069 between 2 and 1000 bp and 16 greater than 1000 bp). Following manual curation and with the help of the Ion Torrent reads and unaligned contigs from the G4ALL and CLC mappings, the number of gaps was reduced to 43, producing a draft assembly with 2 289 075 bp.

To ensure that all the sequenced bases were represented in the assembly, the 443 632 filtered reads were aligned against the draft of the Cp316 genome, identifying 36 444 reads that failed to align. Among these, 19 800 generated 219 contigs via *de novo* assembly and the remaining reads were mapped against genome clusters of *C. pseudotuberculosis* to produce 7687 contigs. All contigs obtained from the *de novo* assembly (7906 contigs) were mapped against the draft genome, and only 139 sequences larger than 100 bp were not mapped. The contigs were inserted in the genome with the help of G4ALL, CLC Genomics Workbench and similarity searches in biological databases, which resulted in the completion of a Cp316 genome assembly containing 2 310 587 bp.

### Genome annotation and pathogenicity islands

**Figure 2:**
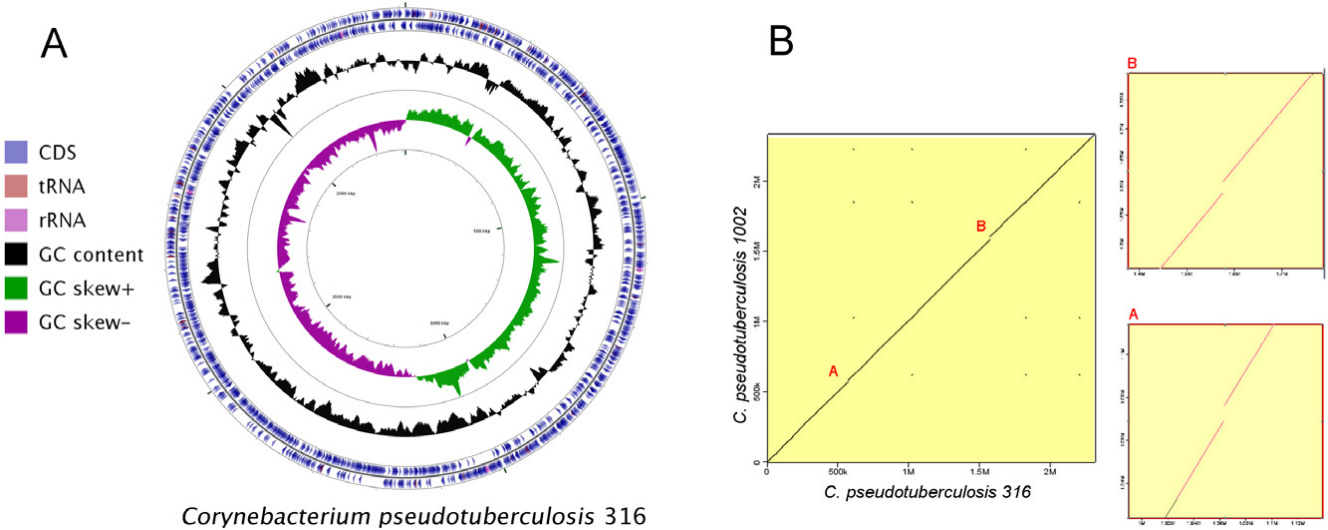
Genome map of *C. pseudotuberculosis* 316 and synteny map between the genome sequences of *Corynebacterium pseudotuberculosis* strains 1002 and 316.A. Genome map of *Corynebacterium pseudotuberculosis* 316 showing common features. CDS (coding sequence); tRNA (transporter RNA); rRNA (ribosomal rRNA).B. Analysis of genome synteny shows two big deletions on *C. pseudotuberculosis* strain 316 when compared with the *C. pseudotuberculosis* strain 1002. Both cases, regions A and B, can be explained through the presence of two pathogenicity islands, PICPs 4 and 5 respectively.

With the prior annotation, we identified more than 400 pseudogenes, many of them due to false frameshifts generated by homopolymers. After manual curation with CLC, only 64 pseudogenes remained (Fig. 2A), which is in agreement with the other strains.

**Figure 3:**
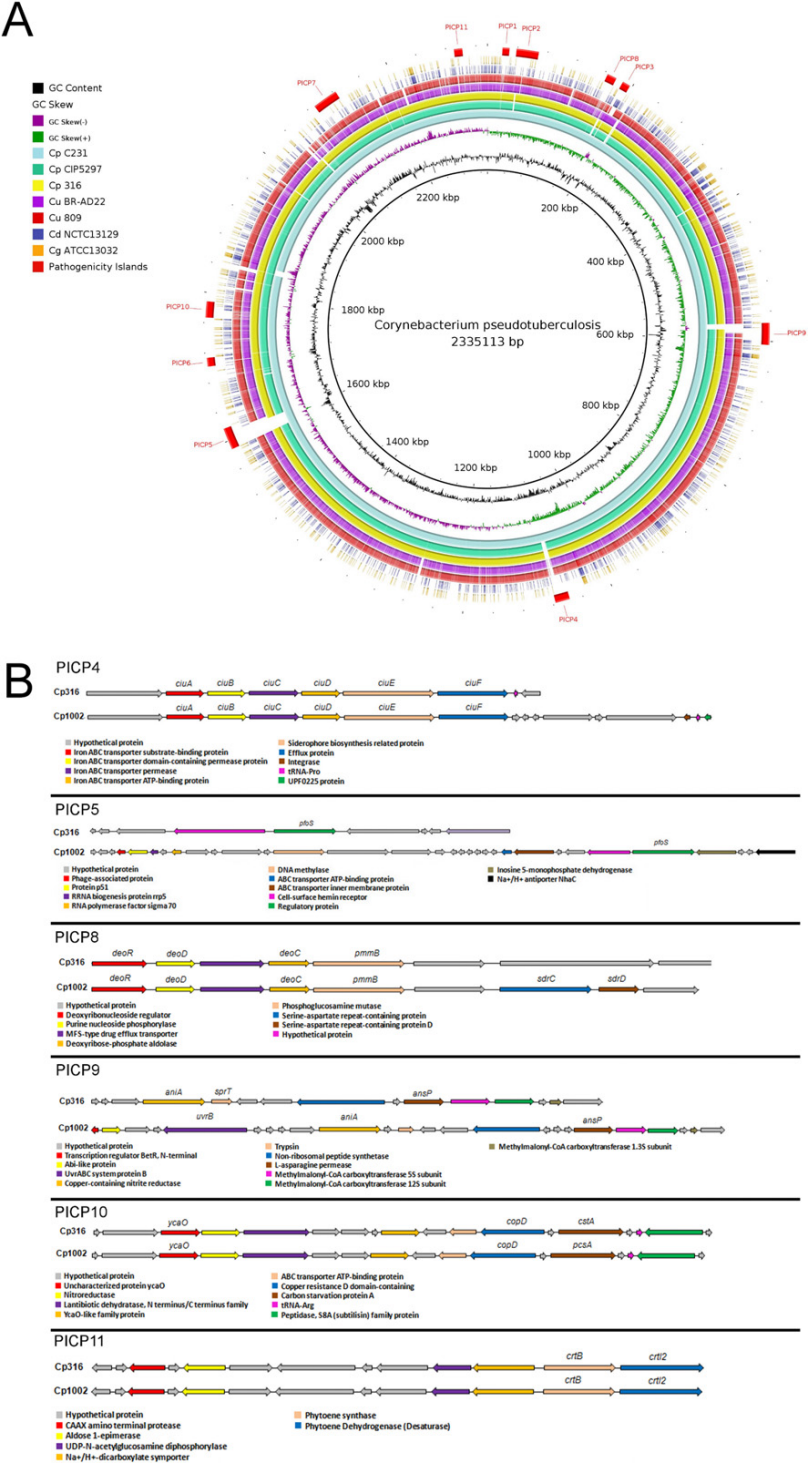
Genomic map comparing strains of *Corynebacterium pseudotuberculosis*, *Corynebacterium ulcerans* and *Corynebacterium diphtheriae*.A. Comparative genomic analyses between: *Corynebacterium pseudotuberculosis* strains 1002, C231, CIP52.97 and 316; *Corynebacterium ulcerans* strains BR-AD22 and 809; *Corynebacterium diphtheriae* NCTC 13129; *Corynebacterium glutamicum* ATCC 13032; and pathogenicity islands identified in *C. pseudotuberculosis*. The figure shows the presence/absence of the pathogenicity islands of *C. pseudotuberculosis* 1002, strain which was also used as reference to create the figure, on the other strains and species.B. Graphical representation of the PAIs 4, 5, 8, 9, 10 and 11 between *C. pseudotuberculosis* 1002 and 316.

The identification of the PAIs of *C. pseudotuberculosis* 316 (GenBank: CP003077) was performed following genome annotation using the Pathogenicity Island Prediction Software (PIPS) (Soares *et al*., 2012). Seven PAIs were identified in *C. pseudotuberculosis* 316 and showed synteny with the PAIs previously described for *C. pseudotuberculosis* strains 1002 and C231 (PICPs 1–7). However, the putative PAIs of *C. pseudotuberculosis* 4 and 5 (PICPs 4 and 5) presented large deletions in strain 316 (Figs S1 and S2), as observed from the synteny map (Fig. 2B), including 30 deleted coding sequences (CDSs) when compared with *C. pseudotuberculosis* strains 1002 and C231 (Fig. 3). Among these 30 CDSs, 22 were annotated as hypothetical proteins and the remaining CDSs presented similarities to an integrase (Cp1002_0990), a phage-associated protein (Cp1002_1448), a p51 protein (Cp1002_1449), rRNA biogenesis protein rrp5 (Cp1002_1450), RNA polymerase factor sigma-70 (Cp1002_1452), DNA methylase (Cp1002_1457) and two ABC transporter ATP binding proteins (Cp1002_1464 and Cp1002_1465). Furthermore, PICP 5 indicated two new CDSs in the *C. pseudotuberculosis* 316 genome that encode hypothetical proteins.

Interestingly, the PIPS program predicted four additional PAIs in *C. pseudotuberculosis* 316 (PICPs 8–11) that were also automatically predicted for *C. pseudotuberculosis* 1002 but were discarded after manual curation. The comparison of *C. pseudotuberculosis* 316 against the genomes of *C. pseudotuberculosis* biovar ovis (1002 and C231) and equi (CIP52.97) strains and those of *Corynebacterium diphtheriae* and *Corynebacterium ulcerans* strains (Fig. 3A) clearly demonstrated that the putative PAIs are located in ‘hotspots’ for horizontal gene transfer, and these regions will be treated as such (Figs S3–S6). Additionally, PICP 9, similarly to PICPs 4 and 5 (Fig. 3B), also has a large deletion when compared with *C. pseudotuberculosis* biovar ovis strains (1002 and C231). The deletions in PICPs 4, 5 and 9 are in agreement with *C. pseudotuberculosis* CIP 52.97, which is also a biovar equi strain, and *C. ulcerans* strains 809 and BR-AD22 (Trost *et al*., 2011). Taken together, these observations corroborate the correct assembly of this genome sequence and may be indicative of the host-specific pattern of the biovars equi and ovis.

## Experimental procedures

### Data collection

The organism *C. pseudotuberculosis* 316 was isolated from the abscess of an American horse in California (USA) and sequenced three times using the Ion Torrent platform (Rothberg *et al*., 2011) with the 314 chip. A total of 160 Mb of sequence was obtained with 69 × coverage. The genome sequence of *C. pseudotuberculosis* FRC41 (GenBank: CP002097), containing approximately 2.3 Mb of genetic information, was used as a reference.

### Data quality treatment

**Figure 4:**
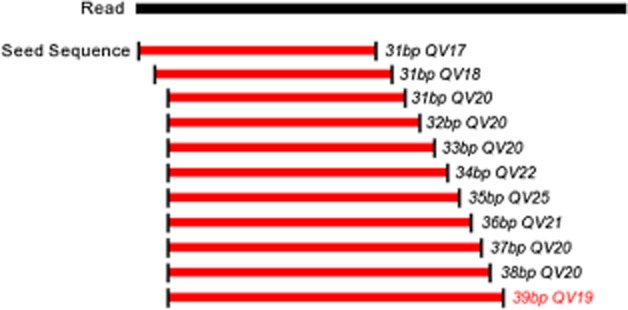
Procedure for the identification of high-quality regions inside Ion Torrent-generated sequences through the search of seeds with medium Phred quality of 20. The long-read version of the Quality Assessment software starts reading the sequence from the first base, using a user-defined window size (31 bp, for example), and walks through the sequence, base by base, until it reaches a region with a mean Phred quality value of 20. After this, the software extends the seed until it reaches either the end of the sequence or a region with a mean Phred quality value lower than 20.

The reads produced by the Ion Torrent platform vary in size, and quality values for the bases are reduced as the 3′ region is approached. Thus, to avoid the disposal of reads due to low-quality bases at the extremities and random trimming of the extremities, a long-read version was developed for the Quality Assessment software (Ramos *et al*., 2011), long-read version (http://sourceforge.net/projects/qualevaluato), which removes the adapters and uses 31 bp seeds to implement a quality filter. The seeds were placed on the first base of the read and moved to the next base until the average quality reached the cut-off value (Phred 20). The seed extension process was initiated from this point until the cut-off value was reached to maximize the use of high-quality regions in the *de novo* assembly (Fig. 4).

### Genome assembly

To produce complementary results in the genome assembly, Velvet 1.0.04 (Zerbino and Birney, 2008) and Edena 3 (Hernandez *et al*., 2008) softwares were used, which utilize the Eulerian Path and overlap–layout–consensus methodologies respectively. The assembly parameter values for the k-mer and coverage cut-off values varied from 29 to 45 and 5 to 15 respectively.

Due to the differently sized reads produced by Ion Torrent and because the Edena3 assembler only accepts sequences of the same size, an in-house script was developed to enable the use of the assembler. Thus, the filtered reads derived from the sequencing were processed by the script, producing new high-quality 50-mer reads (Fig. 1B).

The best set of contigs generated by each of the assemblers was selected and saved in a single file and then subjected to the Simplifier software, which removes redundant sequences from the contig set. The remaining contigs were oriented and ordered using G4ALL, and the BlastN algorithm (Altschul *et al*., 1990) was used to align the contigs against the reference genome of *C. pseudotuberculosis* FRC41 considering a minimum size of 40 bp for the alignment. Those sequences that produced significant hits (alignment size > 39 bp and *E*-value of 1 × 10^−5^) were analysed and extended by the software considering a minimum of 30 bp of overlap between extremities.

Once the contigs were processed through G4ALL, they were inserted into the CLC Genomics Workbench 4.7.2 software and aligned against the genome of *C. pseudotuberculosis* FRC41 to generate a scaffold composed of nucleotide sequences that represent regions not covered by the *de novo* contigs. The coordinates and sizes of the gaps were mapped by an in-house script to be analysed using the CLC Genomics Workbench, in which the alignment of filtered reads was performed against the scaffold. This process was conducted recursively to reduce the number of gaps caused by low-coverage sequencing until it was no longer possible to close the gaps. Thus, version 1 of the genome was produced. This pipeline is presented in Fig. 1.

The filtered reads that failed to align with version 1 of the genome were used for *de novo* assembly using the CLC Genomics Workbench 4.7.2, and contigs were subjected to mapping against the database composed by a set of genomes of *Corynebacterium* available at the NCBI (Table 2 ): *C. diphtheriae* NCTC 13129 (GenBank: NC_002935.2), *Corynebacterium glutamicum* ATCC 13032 (GenBank: NC_006958.1), *C. glutamicum* R chromosome (GenBank: NC_009342.1), *C. pseudotuberculosis* 1002 (GenBank: CP001809.1), *C. pseudotuberculosis* C231 (GenBank: CP001829.1) and *C. pseudotuberculosis* FRC41 (GenBank: NC_014329.1). These genomes were used to generate new contigs to be inserted in strain 316 using the G4ALL software by mapping them against the genome of *C. pseudotuberculosis* 316.

**Table 2:** Descriptions of the species used during the comparative analyses

Genome	Size (bp)	Number of CDS	GC %	rRNA	tRNA	Pseudogene
*Corynebacterium diphtheriae* NCTC 13129	2 488 635	2272	53.5	15	54	47
*Corynebacterium glutamicum* ATCC 13032	3 282 708	3099	53.8	18	60	1
*Corynebacterium glutamicum* R	3 314 179	3080	54.1	18	58	–
*Corynebacterium pseudotuberculosis* 1002	2 335 113	2090	52.2	12	48	53
*Corynebacterium pseudotuberculosis* C231	2 328 208	2091	52.2	11	48	54
*Corynebacterium pseudotuberculosis* FRC41	2 337 913	2110	52.2	12	49	–
*Corynebacterium pseudotuberculosis* 316	2 310 587	2106	52.1	12	49	67

### Assembly validation

CLC Genomics Workbench 4.7.2 software was used to align all filtered reads against the genome of *C. pseudotuberculosis* 316 and therefore identify the set of reads that were not mapped. The unmapped reads were used to establish whether there were regions of the genome that had not been represented, and these regions were subsequently inserted.

### Annotation and frameshift correction

Glimmer software (Delcher *et al*., 1999) was used for genome annotation to predict coding regions. Repetitions in the genome were identified by RepeatScout (Price *et al*., 2005) via a search for similarities against its own database. RNAmmer software (Lagesen *et al*., 2007) was used to predict rRNAs. The protein domain analysis was performed using the Interpro database, which includes several banks of protein domains, motifs and families, and the Interproscan tool (Quevillon *et al*., 2005) was used to increase the reliability of the predictions (Hunter *et al*., 2009).

Frameshifts were identified following the annotations and were mostly generated by the failure to identify homopolymers with Ion Torrent, as cited by Mellmann and colleagues (2011). The frameshifts were corrected through manual curation in the CLC Genomic Workbench, in which the reads produced by the Ion Torrent were aligned against the reference genome of *C. pseudotuberculosis* strain FRC41 (GenBank: NC_014329) and strain 316 of the same organism. The annotated genome sequence of *C. pseudotuberculosis* 316 has been deposited in the GenBank database with Accession Number CP003077.

### Identification of pathogenicity islands

The identification of PAIs was performed using the PIPS program (Soares *et al*., 2012), Artemis Comparison Tool (ACT) (Carver *et al*., 2005) and Blast Ring Image Generator (Alikhan *et al*., 2011). First, the PAIs of *C. pseudotuberculosis* strain 316 were automatically predicted using PIPS, which uses the classical features of PAIs for prediction, i.e. codon usage deviation, atypical G+C content, a high concentration of virulence factors and hypothetical proteins and the presence of transposases and tRNA flanking regions. Following the automatic analysis, the predicted islands were compared with the seven PAIs present in *C. pseudotuberculosis* strain 1002 (GenBank: CP001809) and strain C231 (GenBank: CP001829.1), both from biovar ovis, and strain CIP52.97 (GenBank: CP003061) from biovar equi.

## Supporting information

**Fig. S1.** Comparative analyses of pathogenicity islands of *Corynebacterium pseudotuberculosis*, revealing the deletion of the PICP4 of *C. pseutouberculosis* 316. **Fig. S2.** Comparative analyses of pathogenicity islands of *Corynebacterium pseudotuberculosis*, revealing the deletion of the PICP5 of *C. pseutouberculosis* 316. **Fig. S3.** Comparative analyses of pathogenicity islands of *C. ulcerans* BRAD-22 (PICU6 – at the top), *C. pseudotuberculosis* 1002 (PICP8 – in the middle) and *diphtheriae* NCTC13129 (PICD16-at the botton). **Fig. S4.** Comparative analyses of pathogenicity islands of *C. ulcerans* BRAD-22 (at the top), *C. pseudotuberculosis* 1002 (PICP9 – in the middle) and *C. diphtheriae* NCTC13129 ( at the botton). **Fig. S5.** Comparative analyses of pathogenicity islands of *C. ulcerans* BRAD-22 (PICU10 – at the top), *C. pseudotuberculosis* 1002 (PICP10 – in the middle) and *C. diphtheriae* NCTC13129 (at the botton). **Fig. S6.** Comparative analyses of pathogenicity islands of *C. ulcerans* BRAD-22 (PICU6 – at the top), *C. pseudotuberculosis* 1002 (PICP11 – in the middle) and *C. diphtheriae* NCTC13129 (PICD24 – at the botton).
