# Supplementary material for: Tips and tricks for the assembly of a Corynebacterium pseudotuberculosis genome using a semiconductor sequencer
Source: Microb Biotechnol. 2012 Dec 2;6(2):150–6. doi: 10.1111/1751-7915.12006 (PMC3917457; doi:10.1111/1751-7915.12006)
Supplement: Supplementary file 1 — Fig. S1. Comparative analyses of pathogenicity islands of Corynebacterium pseudotuberculosis, revealing the deletion of the PICP4 of C. pseutouberculosis 316. Fig. S2. Comparative analyses of pathogenicity islands of Corynebacterium pseudotuberculosis, revealing the deletion of the PICP5 of C. pseutouberculosis 316. Fig. S3. Comparative analyses of pathogenicity islands of C. ulcerans BRAD-22 (PICU6 – at the top), C. pseudotuberculosis 1002 (PICP8 – in the middle) and diphtheriae NCTC13129 (PICD16-at the botton). Fig. S4. Comparative analyses of pathogenicity islands of C. ulcerans BRAD-22 (at the top), C. pseudotuberculosis 1002 (PICP9 – in the middle) and C. diphtheriae NCTC13129 ( at the botton). Fig. S5. Comparative analyses of pathogenicity islands of C. ulcerans BRAD-22 (PICU10 – at the top), C. pseudotuberculosis 1002 (PICP10 – in the middle) and C. diphtheriae NCTC13129 (at the botton). Fig. S6. Comparative analyses of pathogenicity islands of C. ulcerans BRAD-22 (PICU6 – at the top), C. pseudotuberculosis 1002 (PICP11 – in the middle) and C. diphtheriae NCTC13129 (PICD24 – at the botton). [file mbt20006-0150-sd1.pdf]

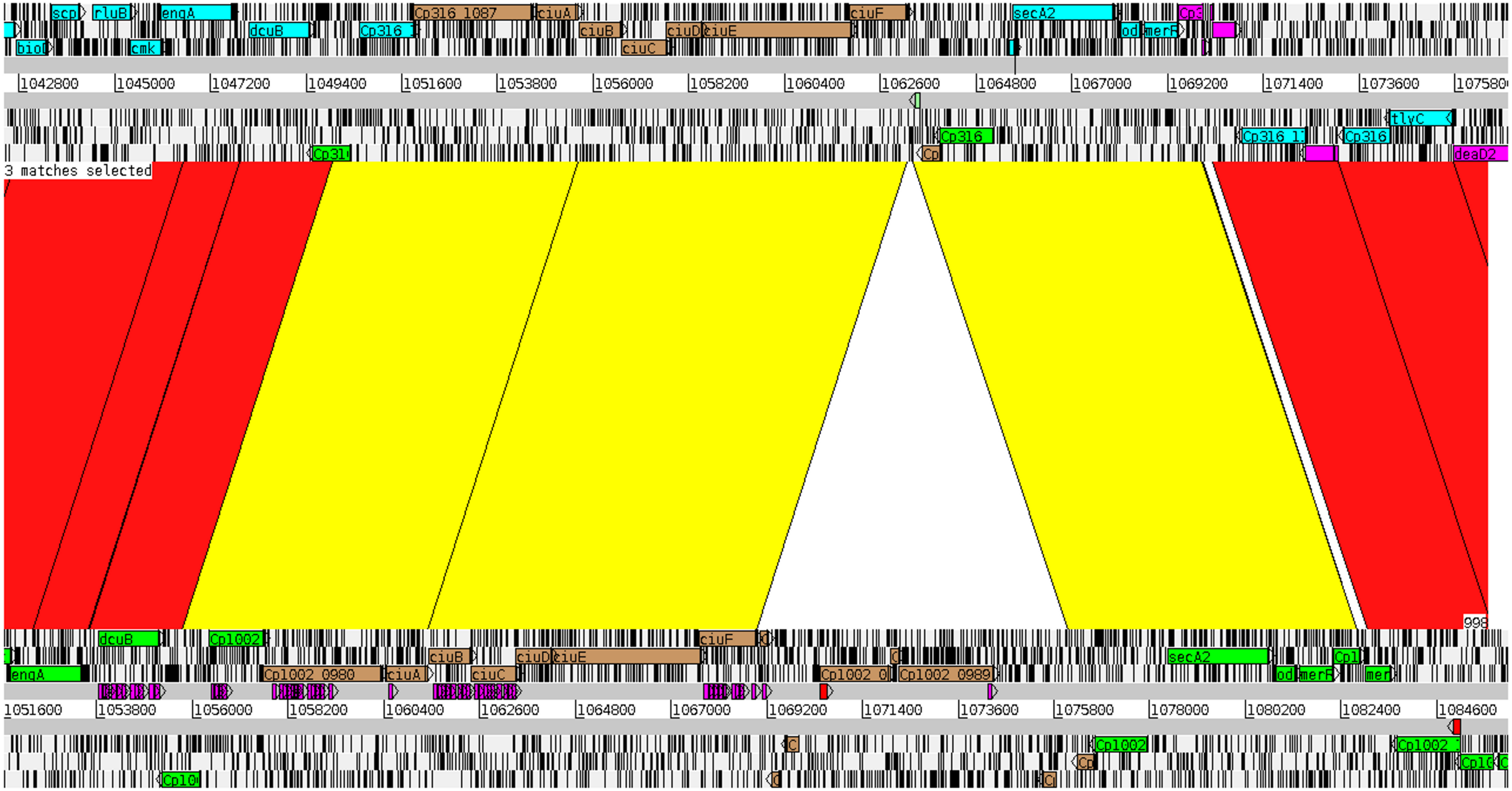

Supplementary Figure S1. Comparative analyses of pathogenicity islands of *Corynebacterium pseudotuberculosis*, revealing the deletion of the PICP4 of *C. pseutouberculosis* 316.

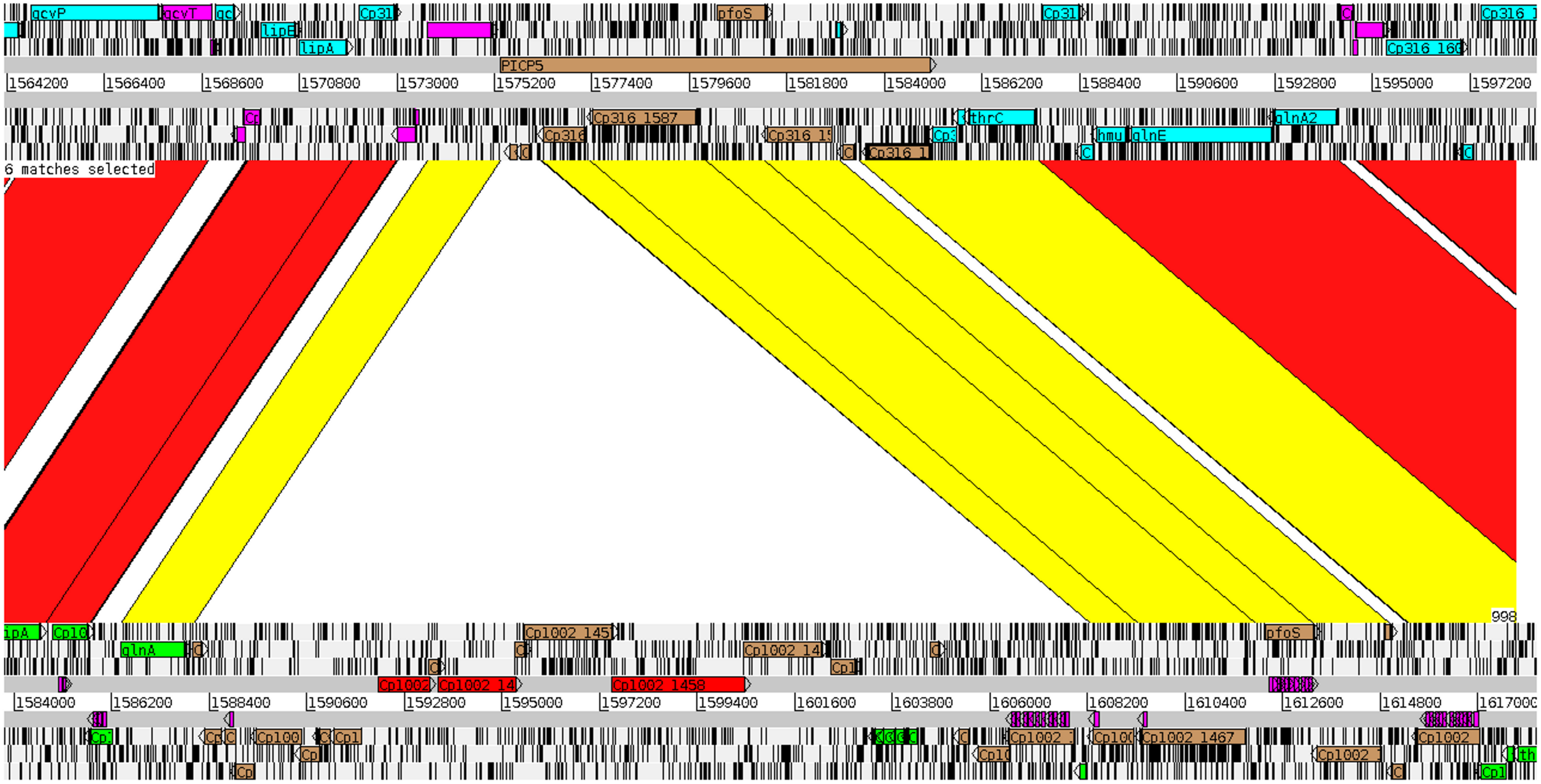

Supplementary Figure S2. Comparative analyses of pathogenicity islands of *Corynebacterium pseudotuberculosis*, revealing the deletion of the PICP5 of *C. pseutouberculosis* 316.

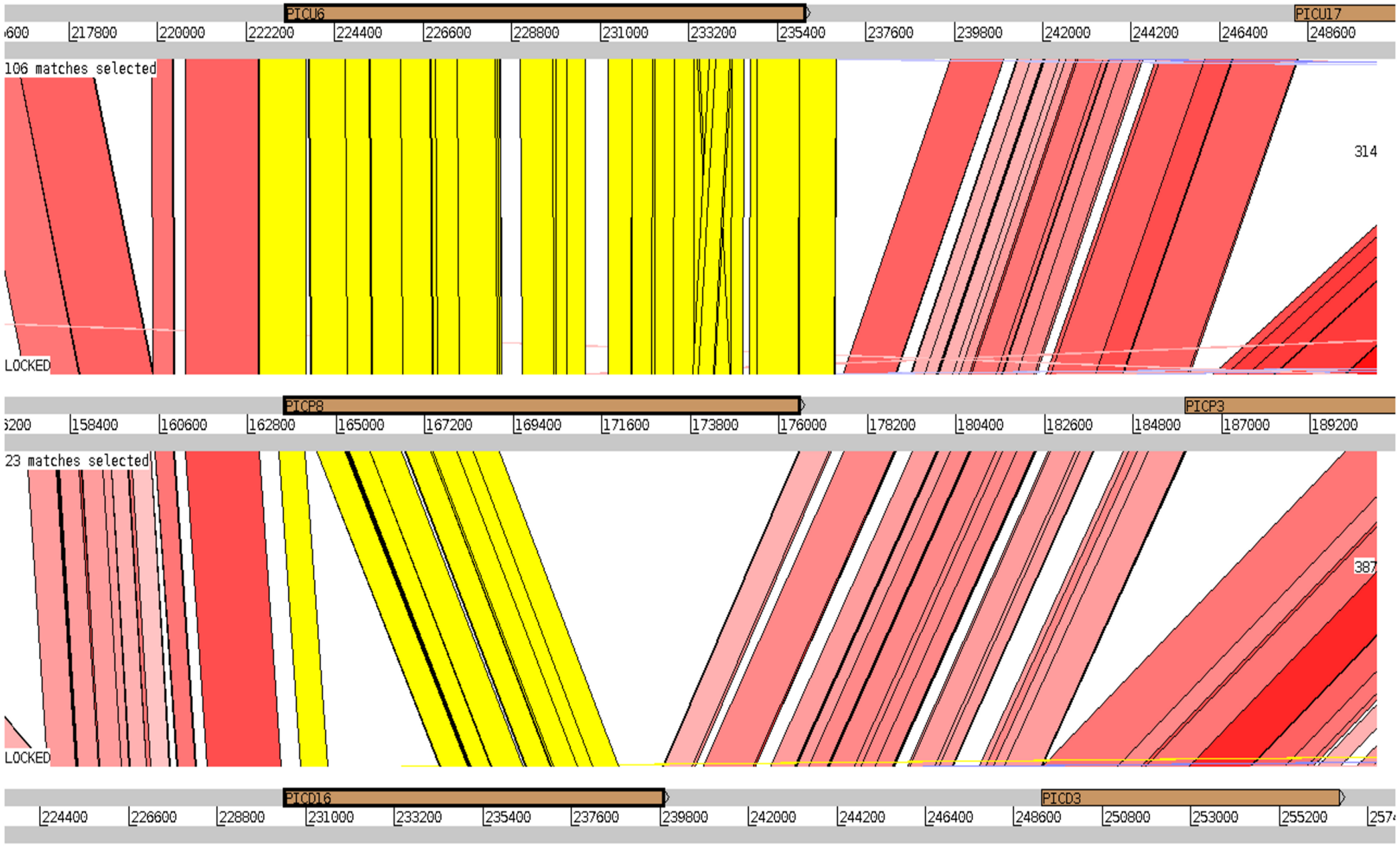

Supplementary Figure S3. Comparative analyses of pathogenicity islands of *C. ulcerans* BRAD-22 (PICU6 – at the top), *C. pseudotuberculosis* 1002 (PICP8 – in the middle) and *diphtheriae* NCTC13129 (PICD16 -at the botton).

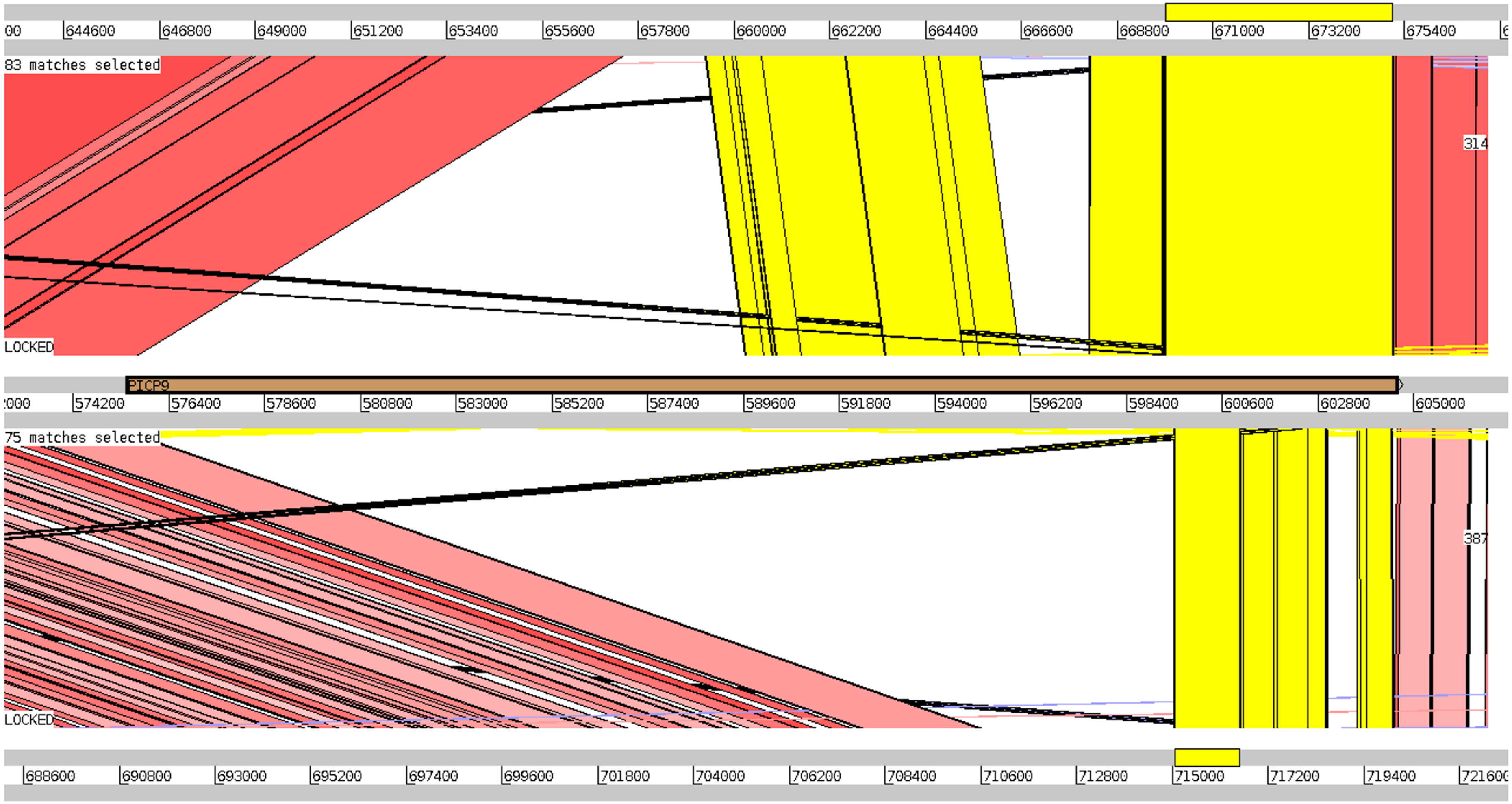

Supplementary Figure S4. Comparative analyses of pathogenicity islands of *C. ulcerans* BRAD-22 (at the top), *C. pseudotuberculosis* 1002 (PICP9 – in the middle) and *C. diphtheriae* NCTC13129 (at the botton).

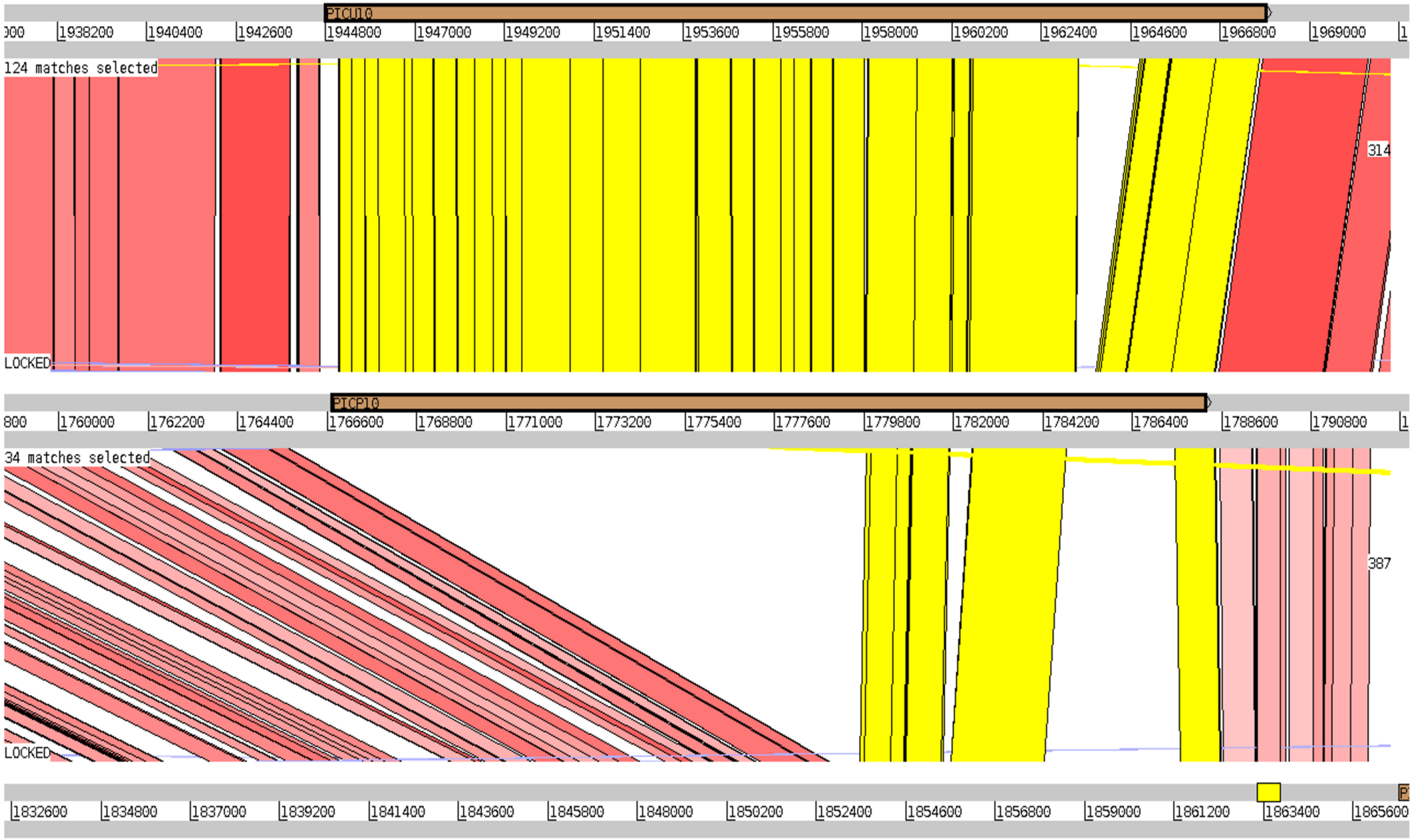

Supplementary Figure S5. Comparative analyses of pathogenicity islands of *C. ulcerans* BRAD-22 (PICU10 – at the top), *C. pseudotuberculosis* 1002 (PICP10 – in the middle) and *C. diphtheriae* NCTC13129 (at the botton).

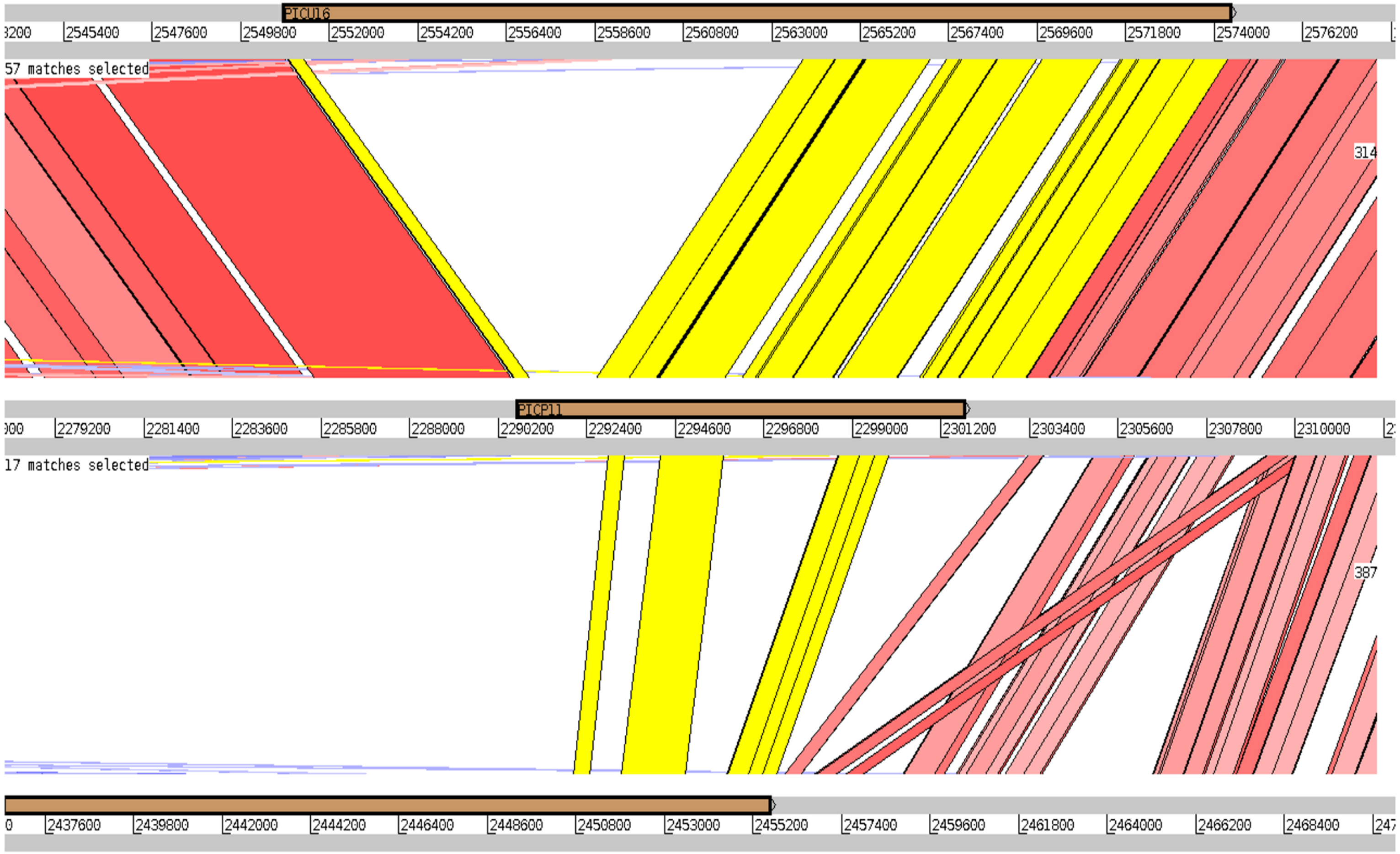

Supplementary Figure S6. Comparative analyses of pathogenicity islands of *C. ulcerans* BRAD-22 (PICU6 – at the top), *C. pseudotuberculosis* 1002 (PICP11 – in the middle) and *C. diphtheriae* NCTC13129 (PICD24 - at the botton).
